# Discovering the sense of touch: protocol for a randomised controlled trial examining the efficacy of a somatosensory discrimination intervention for children with hemiplegic cerebral palsy

**DOI:** 10.1186/s12887-018-1217-5

**Published:** 2018-07-31

**Authors:** Belinda McLean, Misty Blakeman, Leeanne Carey, Roslyn Ward, Iona Novak, Jane Valentine, Eve Blair, Susan Taylor, Natasha Bear, Michael Bynevelt, Emma Basc, Stephen Rose, Lee Reid, Kerstin Pannek, Jennifer Angeli, Karen Harpster, Catherine Elliott

**Affiliations:** 1 School of Adolescent and Child Health, University of Western Australia, Perth, WA Australia; 2 Kids Rehab Department, Perth Children’s Hospital, Perth, WA Australia; 3 Department of Community and Clinical Allied Health, School of Allied Health, La Trobe University, Melbourne, VIC Australia; 4 Neurorehabilitation and Recovery, Stroke Division, Florey Institute of Neuroscience and Mental Health, Melbourne, VIC Australia; 5 School of Occupational Therapy and Social Work, Curtin University, Perth, WA Australia; 6 Cerebral Palsy Alliance, Discipline of Paediatrics and Child Health, The University of Sydney, Sydney, NSW Australia; 7 Telethon Kids Institute, University of Western Australia, Perth, WA Australia; 8 Department of Clinical Research and Education, Child and Adolescent Health Services, Perth, WA Australia; 9 Sir Charles Gairdner Hospital, Perth, WA Australia; 10 Consumer Representative, Perth, WA Australia; 11 Australian e-Health Research Centre, CSIRO, Brisbane, Queensland Australia; 12 Cincinnati Children’s Hospital Medical Center, Cincinnati, Ohio USA

**Keywords:** Cerebral palsy, Upper-limb, Tactile, Sensation, Somatosensory discrimination, Proprioception, Goal directed, Home program

## Abstract

**Background:**

Of children with hemiplegic cerebral palsy, 75% have impaired somatosensory function, which contributes to learned non-use of the affected upper limb. Currently, motor learning approaches are used to improve upper-limb motor skills in these children, but few studies have examined the effect of any intervention to ameliorate somatosensory impairments. Recently, *Sense©* training was piloted with a paediatric sample, seven children with hemiplegic cerebral palsy, demonstrating statistically and clinically significant change in limb position sense, goal performance and bimanual hand-use. This paper describes a protocol for a Randomised Controlled Trial of S*ense© for Kids* training, hypothesising that its receipt will improve somatosensory discrimination ability more than placebo (dose-matched Goal Directed Therapy via Home Program). Secondary hypotheses include that it will alter brain activation in somatosensory processing regions, white-matter characteristics of the thalamocortical tracts and improve bimanual function, activity and participation more than Goal Directed Training via Home Program.

**Methods and design:**

This is a single blind, randomised matched-pair, placebo-controlled trial. Participants will be aged 6–15 years with a confirmed description of hemiplegic cerebral palsy and somatosensory discrimination impairment, as measured by the sense©_assess *Kids*. Participants will be randomly allocated to receive 3h a week for 6 weeks of either S*ense© for Kids* or Goal Directed Therapy via Home Program. Children will be matched on age and severity of somatosensory discrimination impairment. The primary outcome will be somatosensory discrimination ability, measured by sense©_assess *Kids* score. Secondary outcomes will include degree of brain activation in response to a somatosensory task measured by functional MRI, changes in the white matter of the thalamocortical tract measured by diffusion MRI, bimanual motor function, activity and participation.

**Discussion:**

This study will assess the efficacy of an intervention to increase somatosensory discrimination ability in children with cerebral palsy. It will explore clinically important questions about the efficacy of intervening in somatosensation impairment to improve bimanual motor function, compared with focusing on motor impairment directly, and whether focusing on motor impairment alone can affect somatosensory ability.

**Trial registration:**

This trial is registered with the Australian New Zealand Clinical Trials Registry, registration number: ACTRN12618000348257. World Health Organisation universal trial number: U1111–1210-1726.

**Electronic supplementary material:**

The online version of this article (10.1186/s12887-018-1217-5) contains supplementary material, which is available to authorized users.

## Background

Cerebral palsy is the most commonly occurring childhood physical disability, and is an umbrella term covering a variety of aetiologies with a combined prevalence of roughly 2.1 per 1000 live births [1]. It is defined by motor impairment arising from an injury or malformation of the developing brain and is often accompanied by comorbidities such as impairment in sensation, perception, cognition, communication, and behaviour [2]. Hemiplegic CP (HCP; hemiplegia) is the most commonly occurring motor impairment subtype [3] and negatively impacts upper limb function. Recent reports indicate that more than 75% of children with HCP have impaired somatosensory function [4, 5].

Somatosensory function involves the detection, discrimination, and recognition of body sensations [6]. According to the National Institutes of Health toolbox, somatosensation refers to “*all aspects of touch and proprioception that contribute to a person’s awareness of his or her body parts and the direct interface of these with objects and the environment*” p. S41 [6]. This includes body position sense, haptic object recognition, and tactile discrimination [6]. Somatosensation guides motor function in a feed forward manner: the more a child can perceive, the more they explore (move), and the more they can understand and interact with their environment [7, 8]. Ascending somatosensory neural pathways provide tactile and proprioceptive information [9]. By monitoring these forms of information, the central nervous system can adjust signals to descending motor pathways during grasp and associated manipulation of objects [10]. In the upper limbs, both fine motor movements and tool use rely heavily on such feedback [7, 10, 11].

A clear link exists between somatosensory deficits and poor hand function in children with HCP [10, 12]. This was recently demonstrated in a cross-sectional study by Auld et al. [12] where a moderate relationship between tactile function and hand performance was identified. Specifically, haptic object recognition and single point localisation had the greatest influence on unimanual capacity while haptic object recognition and recognition of double simultaneous stimulation had the greatest influence on bimanual function. In this study, impairment in somatosensory function accounted for one third of the variance in motor function [12]. The significant contribution of somatosensation to motor function indicates that therapeutic interventions that target somatosensation may have the potential to improve motor function in children with HCP.

It is recognised that damage to corticomotor tracts and thalamocortical sensory pathways both contribute to upper limb motor impairment in hemiplegia [13–15]. Children with hemiplegia have different patterns of brain activation than typically developing peers during somatosensory tasks [16, 17]. The reorganization of motor pathways is well documented in children with hemiplegia, with a subset showing evidence of persistent and predominant ipsilateral motor pathway control of hand movements [18–27]. Such reorganization is not always functionally advantageous: a noted decline in affected upper limb function is associated with the persistence of ipsilateral pathways in children who sustained injury in late gestation [27]. However, studies investigating somatosensation using magnetoencephalography (MEG), functional magnetic resonance imaging (fMRI) and somatosensory evoked potentials (SEP) of the affected side have demonstrated that activation of the primary somatosensory cortices is often still predominantly in the contralateral hemisphere, and the contralateral pathway still functions, albeit with altered responses [16, 18, 28–32]. This “interhemispheric dissociation” between somatosensory inputs and motor outputs may be a significant contributing factor to the impaired integration of sensorimotor function in a subset of children with hemiplegia [18].

Neuroplastic changes associated with improvement in motor function have been demonstrated following motor learning approaches such as constraint induced movement therapy [33]. Several studies have provided a neurological basis for pursuing somatosensory intervention to improve upper limb function in children with HCP by demonstrating somatosensory pathways are active, albeit disorganised, and therefore possibly treatment responsive [17, 34]. The core principles which inform motor learning approaches to upper-limb therapy are the same as principles of learning dependent neural plasticity such as repetition of a challenging but achievable task, repetitive practice and feedback on performance [35, 36]. It is reasonable to expect that when such principles are applied in a somatosensory intervention, neural plastic changes in somatosensory and related regions of the brain will also be observed. In adult stroke changes have been observed in primary and secondary somatosensory regions and in attention and visual regions in association with better tactile performance [37] and training-facilitated somatosensory recovery [38].

Upper limb function is recognised by experts as a high priority area for treatment of children with hemiplegia [39]. A large body of research has investigated therapeutic interventions and modes of delivery to maximise outcomes for this group of children [40]. Recent research has predominantly focused on improving motor skills via motor learning approaches and has demonstrated that intensive goal-directed treatments have a positive effect on hand function [40]. However, there is limited research into whether reducing developmental non-use and improving bimanual hand function might be more effectively achieved by treating any sensory impairments that are known to contribute to impaired motor function. A recent systematic review of interventions for tactile deficits that may be suitable for children suggested two approaches that were effective in adults post stroke [41]. This study aims to investigate one of those recommended: *transfer enhanced somatosensory discrimination training*, known as *Sense*© training [36].

The principles of *Sense©* training stem from theories of perceptual learning and learning dependent neural plasticity [36]. *Sense©* training involves repeated practice discriminating between graded stimuli in the somatosensory domains: body position sense, haptic object recognition, and tactile discrimination, using specially designed training tasks and perceptual learning [36]. In a randomised controlled trial with cross over control, *Sense©* training was found to improve somatosensory discrimination function in adults (*n* = 50) who were a median of 48 weeks post stroke [36]. In this trial, 69% of stroke survivors at least halved their somatosensory deficits post treatment, and this was maintained at six months’ post treatment. Survivors also achieved transfer of training effects to untrained tasks. Seven training principles are operationalized in the training protocol: selection of specially designed training tasks; goal-directed attentive exploration of sensation without vision; feedback on the accuracy and method of exploration by therapist/vision; calibration of somatosensory perception via vision and/or touch of the unaffected hand; use of deliberate anticipation trials; variety of sensory tasks and practice conditions to facilitate transfer; and repeat and progress, as outlined in the training manual [42] and online video [43]. Sense© is also applied to client-selected activities (occupations), with the aim for the client to learn strategies in how to use somatosensory skills to perform the activity most optimally and to transfer these strategies and skills learnt to untrained activities [42].

Hemiplegia can arise in infants with a variety of neurological pathologies such as white matter injuries, grey matter injuries, malformations of the brain, as well as focal vascular insults (seen in ~ 9% of infants with hemiplegia) and no cerebral pathology that can be identified on imaging in about the same proportion [44]. It cannot be ignored that these aetiologies are highly varied in comparison to adult stroke survivors. Furthermore, most children with HCP have a somatosensory system that has never functioned normally in the extra-uterine world while an adult stroke survivor has received an insult to a previously well-functioning system. Nevertheless it has been suggested that altered structural connectivity is association with severity of deficit and functional recovery [45, 46]. Despite these population differences, pilot work for this study demonstrated that *Sense*© training is feasible with children with HCP and warrants further investigation [47].

During our pilot matched-pairs controlled trial, *Sense*© training was modified to increase suitability for a paediatric population of children with HCP [47]. The principles of training remain the same and children progress through the same levels of graded somatosensory training as adults [36]. To facilitate child engagement with the *Sense*© training, the principles of self-determination theory and family centred service were incorporated into the provision of *Sense© for Kids* training [48, 49]. To improve the relevance of *Sense*© *for Kids* training to children with HCP and their families further modifications were implemented following consumer engagement [50]. Focus groups and interviews were conducted and feedback from children and their families were integrated into changes to *Sense*© *for Kids* training. A consumer representative (EB) also vetted all aspects of this protocol paper and details of the intervention. These changes are aimed at reducing the scheduling demands on families and increasing the education provided to parents. Parent coaching will be used to facilitate maximal carryover of the benefits of therapy into everyday life following the completion of the formal intervention period [51].

Our pilot work suggests that children improve in trained somatosensory domains, motor performance, and in trained occupational tasks [47]. A qualitative investigation of parent and child engagement suggests that improvements were also observed in untrained tasks requiring bimanual function. Improvements following *Sense*© training were maintained six months after training ceased and warrant further investigation with a larger sample [51].

In order to test the efficacy of the *Sense*© *for Kids* training, a “best practice” comparison intervention will be used to provide adequate control for ‘dosage’ and maintain the external validity of this trial [52]. Further, it is considered unethical to withhold potentially effective interventions in controlled comparison conditions. Goal Directed Training delivered via Home Program is an evidence based intervention [40, 53] with a green light on the traffic light system of evidence for children with HCP [54]. Because there are no evidence based somatosensory discrimination interventions for comparison, Goal Directed Training via Home Program will act as our control. Goal Directed Training is a motor learning approach which uses a child’s goals to allow problem solving and indirectly elicit movements needed to complete a task but does not include any direct somatosensory training: it is therefore a ‘best practice’ control intervention incorporating common features of Sense© for Kids training but no direct somatosensory training [55].

## Methods and design

A single blind, matched pair, prospective randomised placebo-controlled trial with parallel groups is proposed comparing the effects of *Sense*© *for Kids* discrimination training with a dose matched, therapist supported Goal Directed Training via Home Program. The primary outcome measure is the sense©_assess *Kids* to assess changes in somatosensory discrimination. The sense©_assess *Kids* measures tactile registration, tactile discrimination, haptic object recognition, and body position sense of the upper-limb in children [56]. The secondary outcome measures are brain imaging including functional magnetic resonance imaging (fMRI) and diffusion MRI to observe central nervous system (CNS) changes in response to intervention, the Assisting Hand Assessment [57] to measure bimanual ability, Goal Attainment Scaling [58] and the Canadian Occupational Performance Measure [59] to monitor change in children’s self-selected goals. This trial has been registered with the Australian New Zealand Clinical Trials Registry, see Table 1 for trial registration data.

**Table 1:** World Health Organisation required trial registry information

Data category	Information
Primary registry and trial identifying number	Australian New Zealand Clinical Trials Registry ACTRN12618000348257
Date of registration in primary registry	8/03/2018
Secondary identifying numbers	None
Source(s) of monetary or material support	Telethon New Children’s Hospital Research Fund
Primary sponsor	Perth Children’s Hospital
Secondary sponsor(s)	University of Western Australia, Curtin University
Contact for public queries	Ashleigh Thornton, PhD Ashleigh.Thornton@health.wa.gov.au
Contact for scientific queries	Belinda McLean, Belinda.McLean2@health.wa.gov.au
Public title	Discovering the sense of touch- somatosensory discrimination training for children with cerebral palsy.
Scientific title	Discovering the sense of touch: A randomised controlled trial examining the efficacy of a somatosensory discrimination intervention for children with hemiplegic cerebral palsy.
Countries of recruitment	Australia
Health condition(s) or problem(s) studied	Cerebral palsy, hemiplegia, impaired tactile discrimination, impaired haptic object recognition, impaired limb position sense
Intervention(s)	Sense© for Kids somatosensory discrimination training; Goal Directed Therapy via Home Program
Key inclusion and exclusion criteria	Inclusion: description of hemiplegic cerebral palsy, somatosensory discrimination impairment as measured by *sense©_assess* kids, aged 6-15 yrs., sufficient concentration to complete assessment. Exclusion: absence of somatosensory impairment fMRI safety exclusion criteria: (metal implants and implantable devices; significant anxiety or behavioural problems; claustraphobia).
Study type	Single-blind randomised control trial
Date of first enrolment	Anticipated 17/09/2018
Target sample size	50
Recruitment status	Not yet recruiting
Primary outcome(s)	*sense©_assess* kids, functional magnetic resonance imaging
Key secondary outcome(s)	Assisting Hand Assessment, Canadian Occupational Performance Measure, Goal Attainment Scaling.

### Interventions

#### Sense© for kids training description

*Sense*© *for Kids* training is a structured and graded intervention program based on *Sense*© somatosensory discrimination training [36, 42]. *Sense*© *for Kids* training will be implemented in this study, as informed by the pilot work that explored the efficacy of *Sense*© somatosensory discrimination training with children with Hemiplegia [47]. *Sense*© *for Kids* training uses principles of perceptual learning and learning dependent neural plasticity to develop somatosensory discrimination capacity in aspects of sensation [60, 61]. The aspects of somatosensation trained are body position sense, haptic object recognition and tactile discrimination. The principles of training are the same as in *Sense*© discrimination training [36] and include active exploration without vision, feedback on accuracy and method of exploration, anticipation trials, calibration with the less affected hand and with vision, repetition and progression from large to finer differences and transfer to occupational tasks. The equipment and training levels are based on the work of Carey et al. [36, 42], see Table 2 for details of the intervention.

**Table 2:** TIDieR Guidelines comparing experimental and control interventions

Item	Experimental intervention	Control intervention
Name	*Sense*© *for Kids*	Goal Directed Training via a Home Program
Why	*Rationale:* The ability to gain a sense of touch and use this information in goal-directed use of the arm and daily activities is supported by theories of perceptual learning and neural plasticity and may be enhanced by addressing somatosensory discrimination functions through intervention [36, 61]. *Sense*© *for Kids* is a structured and graded intervention program based on *Sense*© somatosensory discrimination training [36]. *Theory: Underlying principles of Sense*© • Principles of perceptual learning and learning-dependent neural plasticity inform *Sense*© training principles. *Sense*© is based on seven principles [43], with the theory underlying three core principles outlined. Goal directed attention and deliberate anticipation are important for learning and to facilitate links to somatosensory regions of the brain. Calibration across and within modality improve and create new somatosensory neural connections. Graded progression within and across sensory attributes and tasks are used to facilitate perceptual learning and transfer to novel stimuli [61]. *Sense*© *Essential Elements: as applied to children with cerebral palsy*: • Active exploration without vision of new and known stimuli where the child explores objects/textures/body positions with focus on discriminating differences. • Anticipation is used for previously experienced stimuli; the child knows what to expect to feel and concentrates on attributes of difference without vision. • Calibration occurs within and across modalities with comparison of what is felt by the impaired hand with the less affected hand and with vision. The child matches what they know from visual confirmation and calibration with the less affected hand with their impaired hand. They are prompted to imagine what the sensory stimulus is supposed to feel like based on this knowledge. • Each level of stimulus difference is trained to an accuracy level of 75% correct responses before progressing to a more difficult level of difference. • Transfer to untrained tasks is facilitated by training on a large variety of stimuli and integrating training principles into occupational tasks important the child. Occupational tasks are trained using grading of stimuli, feedback on distinctive features of difference and method of exploration. Additional information can be found in *SENSe: A Manual for Therapists* [42].	*Rationale:* Children with CP learn movements best when they are engaged in practicing real-life activities that are meaningful to them, based on self-identified goals and practice occurs in real-life environments. *Theory: Underlying principles of Goal Directed Training* ▪ Dynamic systems theories of motor control, where movement emerges as a result of the interaction between the person’s abilities, the environment and their goal inform Goal Directed Training. *Underlying principles of Home Programs* ▪ The therapist coaches caregiver and child to build confidence and capabilities ▪ The child and parents are more motivated by self-set goals ▪ Programs set up in the home environment are ecologically valid ▪ Practice is embedded in family routine to permit opportunity for functional practice ▪ Practice of a skill evolves based on performance *Goal Directed Training Essential Elements*: ▪ Caregiver and child set goals about real-life activities the child wants or needs to perform and determines with the therapist which are realistic for intervention. ▪ Examination of the goal-limiting factors at the child, task and environment level. ▪ Changing the task and environment to facilitate child-active independence task performance. ▪ Establishment of a child-active motor practice schedule based on current motor performance, including intense repetition, variation and structured feedback. *Home Program Essential Elements*: ▪ Development of a collaborative partnership characterised by empowerment of parents ▪ Therapist takes on a coaching role in partnership with the parent as the expert in their unique context ▪ Goals are set by the child and parent ▪ A menu of tasks to practice using Goal Directed Training principles are provided to support home practice ▪ Therapists actively support implementation to ensure the program continues to meet family needs and help identify successes [62].
Materials	Therapist: The *Sense*© training kit will be required to train the individual components of sensation. Materials for practice relating to occupational goals will vary depending on the child’s goal e.g. *If the goal is using a knife and fork, food items with varying textures will be required that provide the right level of difference of somatosensory feedback during cutlery use.* A log book will be provided to all families as a reminder to complete home practice incorporating *Sense*© principles into child’s goals, and as an opportunity to increase the challenge as the child improves.	Materials for each child will vary depending on the child’s goal and which elements of the task and environment are being changed to enhance independent performance e.g. *If the goal is catching a tennis ball, materials required may initially include balloons and then light large balls as task modifications to facilitate catching practice at the “just right challenge”*. A log book will be provided to all families as a reminder to practice, and as an opportunity to update the home program as the child improves.
Who	CHILD: Sets functional goals with a clear somatosensory demand in partnership with caregiver if appropriate. THERAPIST: Identifies deficit in somatosensory function and works with child through component training in relevant domains (body position sense, haptic object recognition, tactile discrimination). Supports parent with incorporating *Sense***©** principles into child’s goals. PARENT: Incorporates *Sense***©** principles into child’s goal.	CHILD: Sets functional goals in partnership with caregiver if appropriate. THERAPIST: Determines goal limiting factors and partners with the parent to develop a home-based practice schedule. Also offers coaching and support via home visits PARENT: Carries out the intervention with the child.
How	Home based	Home based
How much	The total dose of *Sense*© for kids will be three hours per week for six weeks with a home visit from a therapist for two hours a week and the family undertaking the remaining one hour of incorporating *Sense*© principles into goal practice. (same dose)	The total dose of this intervention will be three hours per week for six weeks with a home visit from a therapist one hour a week and the family undertaking the remaining accumulative two hours per week of practice. (same dose)
Tailoring	Because children will set their own goals, the activities pertaining to the goal itself may differ but in all other aspects this intervention will remain the same for all participants.	Because children will set their own goals, the activities pertaining to the goal itself may differ but in all other aspects this intervention will remain the same for all participants.
How well	This study will seek to define and measure fidelity of the *Sense*© for Kids intervention for: • Clinician adherence to active ingredients • Intervention receipt There is a home program component of *Sense*© *for Kids* training which focuses on incorporating somatosensory cues into occupational task performance and the facilitation of goal attainment by utilising these somatosensory cues within tasks.	This study will seek to define and measure fidelity of Goal Directed Therapy via Home Programs for: • Clinician adherence to active ingredients • Intervention receipt It is acknowledged that children receiving home programs will have incidental exposure to sensory stimuli through movement and interaction with objects during purposeful activity, however these stimuli will not be emphasised nor will the process of making sense of these somatosensory stimuli.

#### Goal directed home program

This study will follow current best practice descriptions of Goal Directed Training and be delivered using the model home program approach outlined by Novak and Cusick [62]. See Table 2 for details of the intervention.

### Treatment fidelity

Two different types of intervention fidelity will be evaluated in this study. The first will assess clinician adherence to the active ingredients of each intervention protocol. Fidelity checklists containing the active ingredients of the respective intervention protocols have been developed to monitor treatment delivery against a priori criteria (see Additional file 1) [63]. Each criterion will be measured against a four point Likert scale. Adherence to the intervention approach will be determined by the computation of a percentage score [64].

Each intervention session will be video recorded. Assessment of intervention fidelity will include the random selection of 10% of the recorded intervention sessions, and observed by independent third-party reviewers trained in both intervention protocols. A fidelity rating of no less than 80% will be required to consider the intervention delivered to the intervention prototype (i.e. with fidelity).

The second fidelity measure is aimed at intervention receipt [63]. This will be monitored through completion of home practice logs. Participants will be provided with a log book to record practice sessions and note challenges and successes. In addition, parents will be asked to video record their occupational sessions for review, feedback and problem solving with respect to the active ingredients of the respective intervention protocols. These sessions will be reviewed with the treating therapist during home visits weekly. Parents will be asked to use readily available technology such as their mobile phone, if available, for the express purpose of feedback.

#### Ethical considerations

The study will be undertaken at Perth Children’s Hospital, the only dedicated children’s hospital in Western Australia. This study has been prepared in accordance with the principles and mandates set out in the Declaration of Helsinki 2008. Ethics approval has been obtained for this study through Perth Children’s Hospital Human Research Ethics Committees (HREC; ethics number 2014034). Parents and children will be provided with oral and written study information and have the opportunity to have their questions clarified before providing written assent/consent. Informed consent will be sought from primary caregivers and assent from child participants prior to commencement. Because children will be aged eight years and older their assent will be required for them to be enrolled in the study. Participation in this study is voluntary and family’s choices will be respected. Eligibility will be determined during the baseline assessment and randomisation will occur once eligibility has been determined. Children who receive botulinum toxin therapy will continue to receive this treatment, however their baseline assessments will be timed at least twelve weeks post their most recent Botulinum toxin-A injections and these treatments will be recorded.

#### Primary and secondary objectives

Our primary objective is to determine whether *Sense*© *for Kids* training, a somatosensory discrimination intervention, is more effective than placebo (Goal Directed Training via Home Programs) in improving somatosensory discrimination in children with HCP.

The specific hypotheses to be tested are:

- Children receiving six weeks of S*ense*© *for Kids* training will have higher scores on *sense*©*_assess* Kids [56] compared to children who received dose matched goal directed therapy via home program.
- Children receiving six weeks of S*ense*© *for Kids* training will demonstrate changes in fMRI activation of the somatosensory and related processing regions in response to tactile stimulation of the affected limb. Such changes will be greater than any activation changes seen in children who received dose matched goal directed therapy via home program.
- Children receiving six weeks of S*ense*© *for Kids* training will have altered structural connectivity (as assessed with diffusion MRI) of somatosensory processing centres.
- Children receiving six weeks of S*ense*© *for Kids* training will have higher scores on the Assisting Hand Assessment [57] compared to children who received dose matched goal directed therapy via home program.
- Children receiving six weeks of S*ense*© *for Kids* training will have comparable scores on the Goal Attainment Scale [58] and Canadian Occupational Performance Measure [59] compared to children who received dose matched goal directed therapy via home program.

#### Trial design

**Fig. 1:**
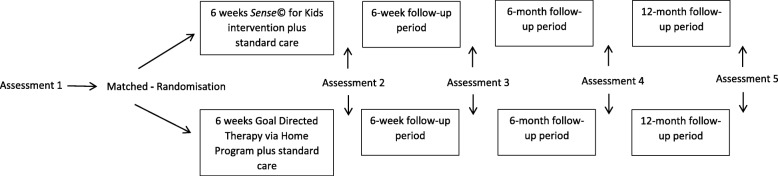
Study design with assessment schedule. Footnote: This figure illustrates the study design and assessment timepoints. Assessment 1 = baseline, assessment 2 = post 6 weeks of intervention, assessment 3 = 6 week follow-up, assessment 4 = 6 month follow-up and assessment 5 = 12 month follow-up. Assessments carried out at each time-point are detailed in Table 2

The Consolidated Standards of Reporting Trials (CONSORT statement 2010) for RCT’s of non-pharmacological treatments will inform this single blind randomised placebo-controlled trial with a matched pair design [65]. Matched pair designs are recommended to reduce covariate effects and strengthen comparisons between groups [66]. Children will be matched on age and composite score [36] on the sense©_assess *Kids* [56]. There will be two arms of this study, *Sense*© *for Kids* training and a dose matched Goal Directed Training via Home Program (Fig. 1). Children will be randomised following baseline assessment to one of these treatment groups. The children in the S*ense*© *for Kids* training group will receive two therapist-directed one-hour treatment sessions per week for six weeks, plus a third hour per week of *Sense*© *for Kids* occupational training carried out by the primary caregiver (who will receive coaching and guidance from the therapist). Children in the Goal Directed Training via Home Program will receive one hour a week of therapist led Goal Directed Training and will undertake a further two hours per week of home practice with primary caregiver support. Differences in therapist directed therapy time exists between these two interventions and reflect the nature of each intervention. The total dose of therapeutic activity is equal.

#### Recruitment

Children will be recruited through the cerebral palsy mobility service at Perth Children’s Hospital, a large state-based tertiary centre.

#### Participants

##### Inclusion criteria

This study will include school aged children and youth:

- With a paediatrician confirmed description of HCP
- Aged 6–15 years
- Who can follow assessment procedure (including fMRI)
- With a confirmed impairment in somatosensory discrimination function as assessed on the sense©_assess_*Kids.*
- Who live within metropolitan Perth, Western Australia

##### Exclusion criteria

This study will not include children and youth who have:

- Upper limb surgery in the 12 months preceding baseline assessments
- MRI contraindications including: metal implants, implantable devices, significant anxiety issues, claustrophobia, or behavioural problems

For children in receipt of Botulinum toxin-A for spasticity management, study commencement will begin 12 weeks after their most recent treatment to allow for Botulinum toxin-A “washout”.

#### Withdrawal

Children and their families are free to withdraw at any time. Any data collected prior to withdrawal will be retained and used for an intention-to-treat analysis.

#### Allocation

Minimisation will be employed to optimise the homogeneity of the two groups [67]. Children will be matched for age (± 6 months) and somatosensory discrimination capacity composite score (mild/moderate/severe). When a child is enrolled to the study without a match for age and somatosensory capacity, that child will be randomly allocated to a treatment group using an online randomisation form by a staff member not otherwise involved in the study. The next child enrolled who is a match for the unmatched participant will be automatically allocated to the alternate group. The process will be repeated for each matched pair; the first member always being allocated at random.

#### Blinding

The families and *treating* therapist(s) will not be blinded to group allocation, but families will be blinded to the study hypotheses. The therapist(s) responsible for *assessment* will be blinded to group allocation. If blinding is broken, this will be noted in the therapist’s treatment or assessment record and reported, where possible a new assessor will be allocated to the participant where unblinding has occurred. To protect the blinding of assessors, participants will be coached not to discuss group allocation with assessors, and interventionists will not discuss study hypotheses with participants.

#### Sample size

To determine the sample size required for this study we used pilot data from seven children with HCP who received the *Sense*© for_Kids intervention [47]. Data from the Wrist Position Sense Test (a component of the sense©_assess_*Kids*, see below) were entered into G* Power [68] and a two tail “Means: difference between two independent groups” power calculation was performed. With an intervention group mean of 15.94 and standard deviation 9.72; and control group mean 25.79 and standard deviation 11.93 the calculated effect size was 0.9052. To detect this effect size, we need 42 subjects (21 in the intervention group and 21 in the control group) to have statistical power of 0.8 at the significance of 0.05. To account for attrition, this study will aim to enroll 50 children, with 25 in each of the control and intervention groups.

#### Retention

Participant retention will be promoted through access to a consistent contact person to address any queries and for coordinating assessment and intervention sessions. As far as possible the booking of assessment and intervention sessions will be flexible to meet participant needs.

#### Study protocol

All outcomes will be measured within two weeks prior to commencement, again within two weeks following completion of intervention, then six weeks, six months and 12 months’ post intervention (± 2 weeks; Table 2). Assessment and intervention will take place in children’s homes or at school, whichever is the most convenient for families, except for MRI assessments which will take place at Perth Children’s Hospital. MRI data will be acquired at all time points, except the 6 weeks follow up.

Table 3 outlines when each outcome measure will be obtained. Time point one is the baseline assessment, time point 2 is at completion of 6 weeks of intervention, time point 3 is 6 weeks’ post intervention completion follow-up, time point 4 is 6 months post intervention completion follow-up and time point 5 is the 12 month post intervention completion follow-up.

**Table 3:** Outcome measures

Outcome measure	Time Point					ICF Domain
	1	2	3	4	5	
Sense©_assess_*Kids*	•	•	•	•	•	Body structure/function
Magnetic Resonance Imaging	•	•		•	•	Brain structure/function
Assisting Hand Assessment	•	•	•	•	•	Activity
Goal Attainment Scaling	•	•	•	•	•	Activity and participation
Canadian Occupational Performance Measure	•	•	•	•	•	Activity and participation

### Outcome measures and procedure

#### Body function and structure

##### Sense©_assess_Kids

The sense©_assess_*Kids* [69] is a suite of tests which measure functional somatosensory discrimination ability. The domains of somatosensation measured by the sense©_assess_*Kids* include the Protective Touch Test [70, 71], the Tactile Discrimination Test [72], the functional Tactile Object Recognition Test [73] and the Wrist Position Sense Test [74]. The Protective Touch Test uses the 4.56 Semmes Weinstein monofilament to test tactile registration at the threshold of protective touch. The Tactile Discrimination Test is a forced choice test of tactile discrimination whereby children need to indicate in a series of presentations which surface out of three is different. The functional Tactile Object Recognition Test is a 14-item test of haptic object recognition with multiple versions in which children are presented with familiar and novel objects out of vision and indicate what they are exploring using a response poster with pictures of all possible items. The Wrist Position Sense Test is a measure of proprioception in which a child’s hand is moved out of vision to 20 positions in random order in the flexion/extension plane of movement of the wrist using a lever and a protractor scale Children indicate where their hand is positioned using a protractor scale immediately above their occluded hand. The sense©_assess_*Kids* has high reliability and normative standards for typically developing children aged 6–15 years [75], and demonstrated construct validity and clinical acceptability for children with CP aged 6–15 years [56, 76].

##### Magnetic resonance imaging

Quantification of central neural change in response to intervention contributes to the understanding of the mechanisms that lead to sustained functional improvements. In this trial, we aim to quantify brain changes that accompany any clinical improvements. To this end, we intend on analysing three types of MRI: *structural MRI*, task-based *functional MRI* (fMRI), and *diffusion MRI* (dMRI).

MR imaging will be conducted on a 3 Tesla Siemens Magnetom Skyra scanner (Siemens, Erlangen, Germany) located at the Perth Children’s Hospital (PCH), Nedlands, Western Australia. Scan types are listed in Table 4 and detailed below. Prior to the initial scan each child will attend an MRI preparation session. This has been demonstrated to improve the success of sedation-free brain MRI scanning in children [77]. The preparation session will include watching a presentation about the MRI experience, familiarisation with the fMRI task (see below) and practice in a mock MRI scanner. On each arrival at the PCH Radiology Department for MRI scans children will be familiarised with the scanning procedure, scanning devices, and receive 5–10 min of practice of the fMRI task. Following the MRI, participants will complete a simple questionnaire regarding the MRI experience including awareness of the stimuli, degree of concentration and comfort.

**Table 4:** MRI scans to be acquired at each of the four time points

	Type	Resolution	Additional Details
T1 MPR	Structural	1 mm iso	3D
T2 FLAIR	Structural	1 mm iso	3D
T2 Blade	Structural		
GRE	field map	3 mm iso	for EPI distortion correction
EPI	Functional	3 mm iso	80 frames
EPI	Diffusion	2 mm iso	8× b = 0 s/mm^2^ 20× b = 1000 s/mm^2^ 60× b = 3000 s/mm^2^

Abbreviations. *MPR* Multiplanar Reformatting, *FLAIR* Fluid Attenuation Inversion Recovery, *GRE* Gradient Echo, *EPI* Echo Planar Imaging

##### Structural MRI

Both high resolution T1 and T2 images will be acquired (see Table 3). The participant will be able to watch a DVD of choice during anatomical sequences. Anatomical reporting will be conducted upon these images by a paediatric neuroradiologist. Baseline MRIs will be classified using the harmonized classification of magnetic resonance imaging, based on pathogenic patterns (MRI classification system or MRICS) proposed by the Surveillance of CP in Europe network [78]. MRI Classification will be documented for each participant and utilised for subgroup data analysis. A paediatrician will meet with the participant and their caregiver to discuss anatomical findings and the primary treating physician will be informed of these results.

##### Functional magnetic resonance imaging

Functional Magnetic Resonance Imaging will be utilised as an indirect measure of neuronal activation in the brain in response to a somatosensory stimulus. Functional MRI utilizes blood-oxygen-level-dependent (BOLD) contrast to indirectly measure neuronal activation in the brain. In neurorehabilitation, fMRI has been utilised to identify, quantify and map cortical activation associated with execution of particular tasks [15]. Functional MRI has also been used in research as a physiological marker of brain plasticity in children with cerebral palsy, and small studies of motor function in children with CP have demonstrated a significant change in task related cortical activation following constraint-induced therapy [79, 80]. Correlation between somatosensory functional impairment post-stroke and central neural changes has been demonstrated using fMRI [36, 81].

Pre-intervention, fMRI activation patterns in response to somatosensory stimulation of both hands will be measured as a baseline, with focus on cortical somatosensory processing centres including primary somatosensory cortex (S1) and secondary somatosensory cortex (S2). Post-intervention fMRI somatosensory task-related activation will be measured and compared to pre-intervention results as an indicator of central neural change in response to therapy. This methodology is supported by literature that indicates that in order to measure neuroplasticity with fMRI, scans should be obtained during a task, both before and after intervention, for at least 20 people per experimental group [82].

In conjunction with the CSIRO, Florey Institute of Neurosciences and Mental Health and La Trobe University, an fMRI protocol [37, 81] has been adapted for use in children with CP. This protocol consists of two acquisitions – one per hand. Each scan will consist of four 30-s ‘touch discrimination’ blocks, each preceded by a 30 s rest block. During touch discrimination blocks, a device is used to present a textured grid to the fingertips in a manner controlled for speed and pressure, alternated with no stimulus. A plastic texture grating is moved side to side across the fingertips of the second, third and fourth digits [37, 81]. Within block, two different plastic texture grids will be delivered, with spacings of 1500 and 3000 μm between the gratings, alternating every five seconds. These texture grids will be presented in a different alternating order every block to maintain attention of the participant. Participants will be instructed to feel and pay attention to the differences between the two textures presented in each block, but to remain still. A screen showing the words ‘FEEL’ or ‘REST’ will be shown to the participant during these respective blocks to cue attending to the stimuli. The pressure of stimulus delivery is calibrated at the commencement of the scan via a weighted pulley system. To control for movement, the participant’s hand rests on a platform with custom openings for the fingertips and is immobilised in the device as the stimulus is moved from side to side under the fingertips. The control ‘REST’ condition of the paradigm is no presentation of the textured grid to the participant’s fingers, though it continues to be moved at a constant speed to the side of the participant’s hand [37, 81]. The participant lies supine throughout.

##### Diffusion magnetic resonance imaging

Diffusion magnetic resonance imaging (dMRI) will be used to investigate brain microstructural changes within pathways delineated using fMRI driven diffusion tractography. dMRI data will be acquired using a multi-shell approach, which includes 8 non-diffusion weighted images, 20 diffusion weighted images at b = 1000s/mm^2^, and 60 diffusion weighted images at b = 3000 s/mm^2^. Correction for susceptibility distortions will be performed using reverse phase-encoded non-diffusion weighted images. Fibre orientation distributions for tractography will be estimated using multi-shell multi-tissue constrained spherical deconvolution [83] implemented in MRtrix software. Fractional anisotropy (FA) will be estimated based on the b = 1000s/mm^2^ shell.

### Activity

The *Assisting Hand Assessment* (AHA) [57] and the *Adolescent Assisting Hand Assessment* (Ad-AHA) [84] are measures of how a child with HCP or brachial plexus palsy uses their involved hand for bimanual activity. The AHA has been found to have good construct validity, excellent test-retest reliability (0.99) and is responsive to change when used to assess children aged 18 months to 12 years [85]. The Ad-AHA utilises the same scoring components as the AHA and has excellent intra-rater (0.97) and test-retest (0.99) reliability [86]. The assessments are conducted as a play session and are video recorded for scoring at a later time [57, 84].

The *Canadian Occupational Performance Measure* (COPM) [59] is a measure of a client’s self-perceived occupational performance over time. The COPM has been found to have good validity and reliability and is responsive to change [87] and has been found to have moderate reproducibility [88].

*Goal Attainment Scaling* (GAS) [58] is a technique used to quantify goals set in a rehabilitation setting. This goal setting technique enables the conversion of measurable goal attainment on a 5-point scale into t-scores which are normally distributed around a mean score of 50 and a standard deviation of 10. The GAS has been found to be a valid and reliable measure of goal attainment [89] with excellent inter-rater reliability (>.90) and satisfactory concurrent validity [90].

### Descriptive measures

To describe our population the following scales and measures will be completed at baseline.

The *Gross Motor Function Classification Scale- Expanded and Revised* (GMFCS-E&R) [91] is a five level scale describing gross motor function for children with cerebral palsy aged 6–12 years and 12–18 years. The GMFCS describes a range of abilities from level I, where children are independently mobile, through to level V where children have limited ability to maintain head and trunk postures and are dependent on wheeled mobility with assistance from others [91].

The *Manual Ability Classification Scale* (MACS) [92] is a five level scale describing the ability of children with cerebral palsy aged 4–18 years to handle objects in everyday activities. The MACS describes a range of manual abilities from level I, where children handle objects easily and successfully, through to level V, where children do not handle objects and are severely limited in their ability to perform simple actions [92].

The *Communication Function Classification Scale* (CFCS) [93] is a five level scale describing the communication ability of individuals with cerebral palsy. The CFCS describes a range of communication abilities from level I, where children are effective senders and receivers with familiar and unfamiliar communication partners, through to level V, where children are seldom effective senders or receivers with familiar communication partners [93].

#### Hypertonia assessment tool

The Hypertonia Assessment Tool (HAT) is a six-item clinical assessment tool used to differentiate between spastic, dystonic and rigid paediatric hypertonia [94]. The HAT allows for standardization of such clinical examination, noting that mixed tone, i.e. both spasticity and dystonia, are present in a large proportion of children with CP [94, 95]. This information will be utilized in subgroup analysis to evaluate whether children with certain hypertonia subtypes demonstrate greater response to intervention than others. In doing so, children with CP can be directed to interventions of greatest efficacy in the future. The HAT has good inter-rater test–retest reliability and validity for the identification of spasticity, and moderate agreement for dystonia [94, 95].

#### Selective control of upper extremity scale

The Selective Control of the Upper Extremity Scale (SCUES) [96] is a measure of selective motor control for the upper limbs. SCUES is a short (< 15 min) video based assessment that is administered by an occupational therapist or physiotherapist. SCUES examines selective motor control for the shoulders, elbow, wrist, and fingers/thumb. The examiner demonstrates a movement, passively moves the child to replicate the movement and determine passive range of motion, then the child replicates the movement. Performance is graded on the presence of mirror movements, movement of additional joints beyond target joint, presence of trunk movement, and the extent to which actual movement is equal to or less than passive range. SCUES has acceptable content validity, intra-rater (> 0.75) and inter-rater (0.72) reliability and construct validity [96].

### Adverse events

Adverse events from intervention and activity based assessment are not anticipated. Adverse events due to imaging aspect of assessment may occur if there is a high degree of anxiety for children about being inside the MRI scanner. Children are not sedated during MRI scans and this research team has developed and piloted a familiarization package to allow children to experience what being inside an MRI scanner is like prior to their consenting to take part in this aspect of the study. The MRI assessment is introduced to children in a standard clinic room with their parents present by the staff members who will be with them on the day of their MRI assessments. All efforts will be made to help children feel comfortable with MRI assessment, however children can withdraw from the MRI assessment if they are experiencing distress and this will not limit their participation in the rest of the study. All adverse events will be reported to the HREC through the chief investigator who will monitor and maintain a register of any adverse events for reporting purposes.

### Statistical methods

As outlined in the CONSORT statement reporting for RCT’s: between group comparisons will be conducted on intention to treat analysis [65]. Missing data arising from incomplete observations and dropouts will be managed using multiple imputations. Multiple imputations is recommended for use in RCT’s because it avoids bias found in last observation carried forward approaches while maintaining power [97].

Summary statistics will be reported for each time point for each group using means and standard deviations. For skewed data, medians and interquartile ranges will be reported.

#### Functional outcome measures

A mixed-effects model with repeated measures will be used to assess within and between group differences. The corresponding baseline measure will be entered as a covariate in the model along with age and somatosensory capacity given these features were used in the randomisation process. The mixed model approach has the advantage of allowing for correlated data (repeated measures) and allows for missing observations within-subject. Model assumptions will be examined and if required transformations applied or non-parametric methods employed. Statistical significance will be set at 0.05.

#### Neuroimaging

##### Functional MRI

First level statistical analyses will contrast blocks (FEEL > REST) on an individual subject basis. Owing to the heterogeneous size and location of brain lesions seen within most cohorts, inter-subject registration (required for voxelwise statistics) may be difficult to perform reliably [98]. We plan to address this by performing region-of-interest analyses that measure the interhemispheric balance of activation between the sensorimotor cortices before and after therapy in the same child. S1, S2, and the dorsolateral prefrontal cortex (as delineated on single-participant templates) will be used. First-level results from all participants will be pooled into an analysis of variance (ANOVA), to investigate (A) changes by time-point, to search for an overall change in brain activation and (B) whether an interaction between time-point and treatment exists.

Task-related fMRI is considered an important neuroimaging modality in researching neuroplasticity. It is however recognised that task-related fMRI presents a “unique set of challenges” [99] that impact data analysis and interpretation. These challenges include but are not limited to: fMRI result variability, inability to distinguish the biological process that underlie changes in activation including alternative explanations such as compensatory activation or strategic shifts, the challenge in presenting task equivalency, and specific challenges in data analysis [99]. Many of these challenges of task-related fMRI are even greater in children with cerebral palsy in comparison to adult populations. As a group, the brain pathology and morphology of children with cerebral palsy is highly heterogeneous and often markedly abnormal owing to the wide range of aetiological processes and the early stage of development at which these processes occur. Additionally, clear relationships between brain structure and a child’s function have been challenging to establish [98]. These factors make standard functional neuroimaging analysis extremely challenging and at risk of limitation in the CP population [100]. Reid et al. make the case that multimodal imaging enables these unique challenges to be addressed and increases the robustness of functional neuroimaging research [99].

This study attempts to reduce the influence of Reid’s cited confounds in a number of ways. First, we have selected long block lengths to reduce effects of abnormal haemodynamic responses. Second, we expect to scan a meaningful number of participants to reduce bias caused by a small number of unusual cases. Third, participants will receive extensive task practice prior to entering the scanner, including in a mock-scanner scenario, reducing differential task anxiety and familiarity between scans. Fourth, we have selected a task with minimal active response required from the participant and should not be substantially more difficult for participants with poorer motor abilities [37, 81]. Fifth, we avoid voxelwise analyses which may be invalidated by pathology. Finally, fMRI analyses will be interpreted in the context of independent clinical scores, measures of cortical thickness, and diffusion measurements of white matter.

##### Traditional diffusion MRI

Traditional diffusion MRI (dMRI) analysis methods make assumptions about brain structure-function relationships that may not hold in the context of significant brain pathology and cortical reorganisation, as occurs in children with CP [99]. To overcome this challenge, surface-based-fMRI guided tractography will be utilised, as previously demonstrated in children with CP [99], to extract thalamocortical tracts.

Mean FA will be taken for thalamocortical tracts at each time point and entered into an ANOVA to test for (A) the effects of time-point, to test for brain changes, and (B) a time-point – treatment interaction, to test whether treatments evoke different degrees of brain change.

##### Data management

Data will be de-identified by way of a code. No personal details will be recorded on any assessment documents. All data will be kept in a locked filing cabinet in a locked room or on a password protected computer in a locked room. Assessments that require identifiable video footage will be stored securely in a locked filing cabinet in a stored room. fMRI imaging will be stored securely on the password protected WA Department of Health electronic radiology imaging system. Scores and data maintained electronically will be on computers that are password protected. No identifiable data will be published. Only the research team will have access to identifiable information. No identifiable data will be published.

To ensure data quality double data entry will be undertaken for a random 10% of the sample. Furthermore, the investigator undertaking data consolidation for statistical analyses will have access to the raw data forms and will be able to review anomalies.

##### Monitoring

This study is subject to annual review by the Perth Children’s hospital HREC. Any changes to the study protocol must first be submitted to the HREC for approval before implementation. Any changes will be communicated to investigators, participants and trial registries by the primary investigators (BM and MBl). Any changes to this protocol will be clearly reported in associated publications. All adverse events and any unanticipated harms will be reported directly to the HREC by the CI who will also maintain a register for the annual review. Because the interventions in this study are activity-based, have been observed to be safe and have a duration of six weeks with no outcomes measured during that period, a data monitoring committee will not be utilised.

##### Dissemination plan

This research will be disseminated by journal publications, workshops, conferences and newsletters to stakeholders, including consumers, of Perth Children’s Hospital. Authorship on publications will be guided by the Telethon Kids Institute Responsible Practice of Research policy.

## Discussion

This study is a phase II comparative clinical trial [101], that builds on the findings of the recently completed phase I feasibility trial, reported by McLean et al. [47]. This comparative clinical trial will make a substantial contribution to our current knowledge base by exploring the efficacy of a somatosensory discrimination approach for children with CP, as well as observing changes in brain function and structure following a somatosensory intervention. This study will also be the first to compare a somatosensory intervention approach in children with HCP with a dose matched evidence based motor function focused intervention, in this case goal directed therapy via home program. It will provide valuable insights into treatment effectiveness and the underlying mechanism for change in the use of somatosensory discrimination training and will add to existing literature concerning the use of home programs. If children gain benefit from somatosensory discrimination training and increase their use of their affected hand and can transfer those skills to novel tasks, such as the children in our pilot work, this will improve functional independence and long-term outcomes for children with HCP. Understanding how a somatosensory approach may impact hand use, a child’s functional independence and self-efficacy will be an important contribution. Further to this, knowing whether a home program alone, without emphasis on sensory stimuli involved in any purposeful activity could have an incidental effect on somatosensory function, will also be an important finding. Results of this study will be disseminated widely through publications, international academic conferences and elsewhere as guided by our consumer representative.

## Additional file

Additional file 1: Treatment Fidelity Checklist. (XLSX 22 kb)

## Abbreviations

Ad-AHA: Adolescent Assisting Hand Assessment
AHA: Assisting Hand Assessment
ANOVA: Analysis of Variance
CFCS: Communication Function Classification System
CNS: Central Nervous System
CONSORT: Consolidated Standards of Reporting Trials
COPM: Canadian Occupational Performance Measure
CP: Cerebral Palsy
dMRI: diffusion Magnetic Resonance Imaging
EPI: Echo Planar Imaging
FA: Fractional anisotropy
FLAIR: Fluid Attenuation Inversion Recovery
fMRI: functional Magnetic Resonance Imaging
GAS: Goal Attainment Scaling
GMFCS-E&R: Gross Motor Function Classification Scale- Expanded and Revised
GRE: Gradient Echo
HAT: Hypertonicity Assessment Tool
HCP: Hemiplegic Cerebral Palsy
HREC: Human Research Ethics Committee
ICF: International Classification of Functioning, Disability and Health
MACS: Manual Ability Classification System
MEG: Magnetoencephalography
MPR: Multiplanar Reformatting
MRI: Magnetic Resonance Imaging
PCH: Perth Children’s Hospital
SCUES: Selective Control of the Upper Extremity Scale
SEP: Somatosensory evoked potentials
